# Corneal confocal microscopy: a novel biomarker of small fibre neuropathy in SLE

**DOI:** 10.1136/lupus-2025-001645

**Published:** 2025-10-23

**Authors:** Miral H Gharib, Georgios Ponirakis, Soha O M Dafaalla, Hoda Gad, Einas Elgassim, Hadeel B Zaghloul, Adnan Khan, Ioannis N Petropoulos, Gulfidan Bitirgen, Samar Al Emadi, Rayaz A Malik

**Affiliations:** 1 Hamad Medical Corporation, Doha, Qatar; 2 Weill Cornell Medicine - Qatar, Doha, Qatar; 3 Department of Ophthalmology, Necmettin Erbakan University, Meram, Turkey

**Keywords:** Systemic Lupus Erythematosus, Autoimmune Diseases, Quality of LIfe

## Abstract

**Objective:**

Small fibre neuropathy (SFN) is an under-recognised complication of SLE that contributes to chronic pain and reduced quality of life. We assessed the utility of corneal confocal microscopy (CCM) for identifying small fibre damage in SLE in relation to disease activity, neuropathic pain and quality of life.

**Methods:**

Participants with SLE and healthy controls underwent CCM to quantify corneal nerve fibre density (CNFD), corneal nerve branch density (CNBD), corneal nerve fibre length (CNFL), corneal nerve fibre tortuosity (CNFT), inferior whorl length (IWL), Douleur Neuropathique 4 (DN4) Score, vibration perception threshold (VPT) and sudomotor function.

**Results:**

Participants with SLE (n=59; age 38.6±9.6 years; mean Systemic Lupus Erythematosus Disease Activity Index Score 3.4±4.2) had significantly lower CNBD (41.5±21.3 vs 72.1±29.4 branches/mm², p=0.0001) and CNFL (18.5±4.3 vs 24.2±4.4 mm/mm², p=0.0001) but comparable CNFD (31.7±7.1 and 34.0±6.9 fibres/mm^2^, respectively, p=0.25), CNFT (15.0±4.0 and 14.3±3.1, respectively, p=0.55), and IWL (38.5±8.0 and 35.6±5.9 mm/mm^2^, respectively, p=0.16) compared with healthy controls (n=17). Patients with SLE had a DN4 Score of 3.5±2.5 and elevated VPT (4.1±3.3 vs 2.8±0.7 V, p<0.01) but comparable sudomotor function of the hands and feet (p=0.28–0.42). Active SLE was associated with a lower CNBD/CNFD ratio (p<0.05). Patients with SLE associated with sustained neuropathic pain (17.2%) had significantly lower CNFD, CNFL and IWL than those with transient (p<0.05–0.0001) and recurrent (p<0.05–0.01) pain but comparable VPT (p=0.27) and sudomotor function (p=0.14). Reduced CNFL was associated with bodily pain, affecting quality of life (p<0.05).

**Conclusion:**

This study demonstrates that CCM detects peripheral neurodegeneration in patients with SLE, which relates to disease activity, sustained neuropathic pain and quality of life. CCM may serve as a rapid non-invasive neuroimaging technique to identify SFN in SLE.

**WHAT IS ALREADY KNOWN ON THIS TOPIC**

- Small fibre neuropathy (SFN) is a recognised yet underdiagnosed complication in SLE, contributing to chronic pain and impaired quality of life.
- Traditional diagnostic tools for SFN are either invasive or lack sensitivity, creating a need for better non-invasive biomarkers.

**WHAT THIS STUDY ADDS**

- This study demonstrates that corneal confocal microscopy (CCM) can detect nerve fibre changes associated with SLE disease activity, neuropathic symptoms and quality of life.

**HOW THIS STUDY MIGHT AFFECT RESEARCH, PRACTICE OR POLICY**

- CCM shows potential as a rapid, non-invasive technique to assess small fibre involvement in SLE.
- It may aid early identification of patients at risk for neuropathic pain, paving the way for timely interventions and improved quality of life.

## Introduction

 SLE is a chronic multisystem autoimmune disease with clinical features ranging from mild manifestations such as skin rashes, oral ulcers and non-erosive arthritis, to life-threatening complications such as lupus nephritis and haematological or neuropsychiatric disorders.^1^ A key feature of SLE is neuropsychiatric SLE (NPSLE), which has been formally classified into 19 distinct syndromes by the American College of Rheumatology (ACR).^1 2^ Indeed, 80%–90% of patients with SLE will develop neuropsychiatric manifestations that can severely affect their quality of life and are associated with poor long-term outcomes.^3^,^5^ In a study of 2097 patients with SLE, 5.9% had peripheral neuropathies, of whom 17.1% had small fibre neuropathy (SFN).^6^ Although SFN is a common cause of painful neuropathy and reduces quality of life, it is underdiagnosed and not included in the NPSLE criteria.^5^,^10^ Furthermore, the diagnosis of SFN is challenging. Traditional methods such as nerve conduction studies assess large fibres, whereas skin biopsy can be used to diagnose SFN, although it is invasive. Delay in SFN diagnosis can contribute to disease refractoriness;^5^,^12^ consequently, there is an urgent need for rapid and reliable methods to diagnose and monitor SFN in patients with SLE. Corneal confocal microscopy (CCM) is a non-invasive ophthalmic imaging technique, first conceptualised by Marvin Minsky in 1955 to image neural networks in living tissue.^13^ More recently, our group and others have explored the use of CCM as a non-invasive ophthalmic neuroimaging technique to identify diabetic peripheral neuropathy.^14^,^17^ CCM has subsequently been used to study other peripheral neuropathies,^18^,^20^ and central neurodegenerative diseases, including dementia,^21^ Parkinson’s disease,^22 23^ stroke^24^ and multiple sclerosis.^25^,^27^ Additionally, CCM has been used to identify nerve degeneration associated with long covid^28^ and other immune-mediated neuropathies, including Sjögren’s syndrome^29 30^ and Behçet’s disease.^30 31^ In ophthalmology, CCM is also widely used for other indications, including the diagnosis of infectious keratitis, particularly *Acanthamoeba* and fungal infections, through visualising cysts, filaments, and stromal or endothelial defects.^32^

The potential of CCM to serve as a biomarker for SFN in SLE is substantial, given the non-invasive nature of the technique and its ability to detect early nerve damage, as shown in our previous pilot study that established an initial understanding of how CCM can be used to detect SFN in patients with SLE and identify considerable nerve and immune cell changes linked to disease activity.^33^ Building on these insights, the current expanded study with a larger cohort aimed to assess whether CCM can be used to detect neurodegeneration in SLE in relation to disease activity, neuropathic pain and quality of life.

## Methods

### Study design and population

This cross-sectional single-visit study included adult patients with SLE and healthy controls from the Rheumatology Clinic of Hamad Medical Hospital in Qatar and was conducted from 3 October 2021 to 31 December 2022. The study was conducted in compliance with the Declaration of Helsinki, Good Clinical Practice guidelines, and laws and regulations of the Ministry of Public Health, Qatar. All patients provided written informed consent prior to inclusion.

Adult patients with SLE were recruited by an experienced rheumatologist based on the revised ACR SLE classification criteria.^34^ Exclusion criteria included a history of ocular surgery or trauma, corneal pathology, contact lens use, allergy to local anaesthetics, diabetes or any systemic disease that could cause neuropathy (eg, vitamin B_12_ deficiency and hypothyroidism), in addition to a Schirmer Test Score of ≤5 mm in 5 min. Healthy controls were recruited from Hamad General Hospital and Weill Cornell Medicine in Qatar.

Healthy controls were age-matched to the SLE group but recruited in smaller numbers, as they served mainly as a reference group for CCM measures. Laboratory tests for SLE included complete blood count, serum creatinine, antinuclear antibodies, anti-double-stranded DNA (anti-dsDNA), extractable nuclear antigen panel, antiphospholipid antibodies (lupus anticoagulant and IgG and IgM), anticardiolipin antibodies, complement components C3 and C4, erythrocyte sedimentation rate and urine protein-to-creatinine ratio. Blood tests were also performed to exclude other causes of neuropathy, including glycated haemoglobin A1c (HbA1c), serum vitamin B_12_, folate, free thyroxine and thyroid-stimulating hormone levels.

Disease activity was assessed using the Safety of Estrogens in Lupus Erythematosus National Assessment–Systemic Lupus Erythematosus Disease Activity Index (SELENA–SLEDAI), which classifies activity as follows: none (0), mild (1–5), moderate (6–10), high (11–19) and very high (≥20).^35^

### Corneal confocal microscopy

CCM was performed using the Rostock Corneal Module/Heidelberg Retinal Tomograph. After anaesthetising the cornea with 0.4% oxybuprocaine hydrochloride and applying Viscotears as a coupling agent, participants positioned their chin on the CCM and fixated on a light for central and inferior corneal examinations. The cornea was scanned in section mode, following a standardised image selection protocol.^36^ CCM images were extracted at a separate time by an investigator who was blinded to the patient diagnosis. Subsequently, three to five high-quality sub-basal nerve plexus images from the central and inferior whorl regions were analysed using CCMetrics software^37^ to quantify the corneal nerve fibre density (CNFD; fibres/mm²), corneal nerve branch density (CNBD; branches/mm²), corneal nerve fibre length (CNFL; mm/mm²), inferior whorl length (IWL, mm/mm^2^), CNBD/CNFD ratio and IWL/CNFL ratio.

### Peripheral neuropathy assessment

The vibration perception threshold (VPT) was measured using a neurothesiometer on the pulp of the large toe and repeated three times; the average value was recorded.^38^ Sudomotor function was measured using the SudoScan, which assesses electrochemical skin conductance in the palms and soles, reflecting sympathetic autonomic nerve function.^39 40^

### Neuropathic symptoms and quality of life

Neuropathic symptoms were assessed using the Douleur Neuropathique 4 (DN4) Questionnaire, which was also used to define neuropathic pain as demonstrating a score of ≥4. The McGill Pain Questionnaire was used to assess neuropathic pain patterns, classifying pain as sustained, recurrent or transient according to participant responses.^41^ Quality of life was assessed using the 36-Item Short Form Health Survey (SF-36) Questionnaire, which covers eight domains of physical and mental health, with higher scores reflecting better health status.^42^

### Statistical analysis

Since this was an exploratory study and there were no robust previous data, a power calculation to determine sample size was not possible. Continuous variables are presented as mean±SD, and categorical variables are expressed as percentages. Comparisons between the SLE group and healthy controls were performed using independent t-tests. Categorical variables were compared using the χ^2^ test. Subgroup comparisons between the controls; active and inactive SLE groups; and SLE groups with transient, recurrent and sustained pain were assessed using one-way analysis of variance, followed by post hoc pairwise least significant difference comparisons.

Associations between corneal nerve measurements and VPT as independent variables and SF-36 quality-of-life domains as dependent variables were examined using multiple linear regression models. Demographic, clinical and laboratory variables were screened in bivariate analyses against each SF-36 domain. Variables with p<0.05 were entered as confounders in the multiple linear regression model for that domain. Physical functioning was adjusted for age and haemoglobin, whereas all other domains were adjusted for haemoglobin only. Statistical significance was defined as p<0.05. Statistical analyses were performed using SPSS software (V.26; IBM Corp, Armonk, New York, USA).

## Results

### Clinical characteristics

Participants with SLE (n=59) and healthy controls (n=17) with comparable ages (38.6±9.6 years and 34.0±7.5 years, respectively, p=0.07) were included (table 1). In the SLE group, 94.9% were women, 62.7% had active SLE, 49.2% had neuropathic pain, 10.2% had Sjögren’s disease and 28.8% had nephritis. The mean SLEDAI Score and erythrocyte sedimentation rate were 3.4±4.2 and 19.7±13.9 mm/hour, respectively. Additionally, 36.2% had positive anti-dsDNA antibodies, 22.4% had low C3 levels and 19.0% had low C4 levels. Of the patients with SLE, 96.6% were on antimalarial drugs, 62.1% on immunosuppressants (30.3% on azathioprine and 28.8% on mycophenolate) and 44.1% on steroids. The mean duration of SLE was 8.7±6.6 years (1–1.5 years in 16.4%, 2–9 years in 47.3% and ≥10 years in 36.4%). The body mass index, HbA1c, and systolic and diastolic blood pressure values were comparable between the two groups (p=0.31–0.76).

**Table 1:** Comparison of clinical, neuropathy and quality-of-life characteristics between patients with SLE and healthy controls

	Controls (n=17)	Patients with SLE (n=59)	P value
Age, years	34.0±7.5	38.6±9.6	0.07
Female sex, n (%)	13 (76.5)	56 (94.9)	<0.05
Active SLE, n (%)	N/A	37 (62.7)	N/A
SLEDAI, Score	N/A	3.4±4.2	N/A
Erythrocyte sedimentation rate, mm/hour	N/A	19.7±13.9	N/A
Anti-dsDNA level (IU/mL)	N/A	25.6±50.4	N/A
C3 level (g/L)	N/A	5.0±21.2	N/A
C4 level (g/L)	N/A	1.2±5.7	N/A
Neuropathic pain, n (%)			
Transient	N/A	12 (20.7)	N/A
Recurrent	N/A	36 (62.1)	N/A
Sustained	N/A	10 (17.2)	N/A
Disease duration, n (%)			
1–1.5 years	N/A	9 (16.4)	N/A
2–9 years	N/A	26 (47.3)	N/A
≥10 years	N/A	20 (36.4)	N/A
Antimalaria medication, n (%)	N/A	57 (96.6)	N/A
Azathioprine immunosuppressants, n (%)	N/A	18 (30.3)	N/A
Mycophenolate immunosuppressants, n (%)	N/A	17 (28.8)	
Steroids, n (%)	N/A	26 (44.1)	N/A
CNFD, fibres/mm^2^	34.0±6.9	31.7±7.1	0.25
CNBD, branches/mm^2^	72.1±29.4	41.5±21.3	<0.0001
CNFL, mm/mm^2^	24.2±4.4	18.5±4.3	<0.0001
IWL, mm/mm^2^	35.6±5.9	38.5±8.0	0.16
CNFT	14.3±3.1	15.0±4.0	0.55
CNBD/CNFD ratio	2.07±0.57	1.30±0.62	<0.0001
IWL/CNFL ratio	1.50±0.27	2.16±0.64	<0.0001
DN4 Questionnaire, Score	0±0	3.5±2.5	<0.0001
Neuropathic pain, n (%)	0 (0)	29 (49.2)	<0.0001
VPT, V	2.8±0.7	4.1±3.3	<0.01
Sudomotor function of the hands, ESC	67.4±15.5	63.0±19.9	0.42
Sudomotor function of the feet, ESC	76.5±4.3	71.8±16.0	0.28
Physical functioning, %	94.4±11.2	75.8±20.8	<0.0001
Role limitations due to physical health problems, %	92.6±14.7	59.7±38.6	<0.0001
Role limitations due to emotional problems, %	98.0±8.1	60.5±41.7	<0.0001
Energy/fatigue, %	60.4±11.9	50.0±16.0	<0.01
Emotional well-being, %	73.4±14.1	65.0±22.9	0.07
Social functioning, %	96.3±9.6	68.4±27.6	<0.0001
Bodily pain, %	90.0±15.4	64.9±23.0	<0.0001
General health, %	74.1±16.9	57.4±21.9	<0.01
Systolic blood pressure, mm Hg	120.5±10.8	118.5±14.4	0.59
Diastolic blood pressure, mm Hg	76.9±17.3	74.5±10.2	0.46
BMI, kg/m^2^	25.9±5.0	27.4±4.8	0.31
HbA1c level, %	5.3±0.3	5.3±0.4	0.76

Variables summarised as mean±SD were compared using the independent t-test.

BMI, body mass index; CNBD, corneal nerve branch density; CNFD, corneal nerve fibre density; CNFL, corneal nerve fibre length; CNFT, corneal nerve fibre tortuosity; DN4, Douleur Neuropathique 4; dsDNA, double-stranded DNA; ESC, electrochemical skin conductance; HbA1c, glycated haemoglobin A1c; IWL, inferior whorl length; N/A, not applicable; SLEDAI, Systemic Lupus Erythematosus Disease Activity Index; VPT, vibration perception threshold.

### Comparison of active and inactive SLE

Compared with the inactive SLE group, the active SLE group (62.7%) had significantly higher anti-dsDNA levels (39.0±59.3 vs . 2.0±1.6 IU/mL, p=0.0001) and non-significant trends for lower C3 (p=0.06) and C4 (p=0.09) levels (table 2). Steroid use was significantly higher in the active SLE group than in the inactive SLE group (54.1% vs 27.3%, p<0.05). Immunosuppressant use was higher in the active SLE group than in the inactive SLE group, although not significantly (70.3% and 47.6%, respectively, p=0.09).

**Table 2:** Comparison of clinical, neuropathy and quality-of-life characteristics between inactive and active SLE

	Controls	Patients with inactive SLE	Patients with active SLE	P value
N (%)	17	22 (37.3)	37 (62.7)	N/A
Age, years	34.0±7.5	40.4±10.6†	37.5±9.0	0.25
Anti-dsDNA level (IU/mL)	N/A	2.0±1.6	39.0±59.3	0.001
C3 level (g/L)	N/A	2.9±9.3	0.2±0.1	0.06
C4 level (g/L)	N/A	12.1±34.7	1.0±0.3	0.09
Immunosuppressants, n (%)	N/A	10 (47.6)	26 (70.3)	0.09
Steroids, n (%)	N/A	6 (27.3)	20 (54.1)	<0.05
CNFD, fibres/mm^2^	34.0±6.9	30.8±7.3	32.2±7.0	0.47
CNBD, branches/mm^2^	72.1±29.4	45.5±17.5**	39.1±23.1***	0.31
CNFL, mm/mm^2^	24.2±4.4	18.8±4.0***	18.4±4.5***	0.75
IWL, mm/mm^2^	35.6±5.9	37.7±9.0	39.0±7.4	0.54
CNFT	14.3±3.1	15.8±4.7	14.5±3.4	0.20
CNBD/CNFD ratio	2.07±0.57	1.53±0.65*	1.17±0.56***	<0.05
IWL/CNFL ratio	1.50±0.27	2.05±0.54*	2.23±0.69***	0.27
DN4 Questionnaire, Score	0.0±0.0	4.1±2.4***	3.2±2.5***	0.12
VPT, V	2.8±0.7	3.4±1.5	4.4±4.0	0.21
Sudomotor nerve function of the hands, ESC	67.4±15.5	67.4±17.5	60.4±21.0	0.18
Sudomotor nerve function of the feet, ESC	76.5±12.7	74.7±14.0	70.1±17.0	0.27
Physical functioning, %	94.4±11.2	74.5±18.1*	76.5±22.5*	0.71
Role limitations due to physical health problems, %	92.6±14.7	73.9±30.4	51.4±40.8***	0.01
Role limitations due to emotional problems, %	98.0±8.1	71.2±37.5†	54.1±43.3***	0.09
Energy/fatigue, %	60.4±11.9	52.8±14.4	48.3±16.8*	0.28
Emotional well-being, %	73.4±14.1	70.5±17.8	61.7±25.0	0.12
Social functioning, %	96.3±9.6	72.2±21.8*	66.2±30.6***	0.38
Bodily pain, %	90.0±15.4	64.8±22.9**	65.0±23.4***	0.97
General health, %	74.1±16.9	62.7±17.9	54.2±23.6*	0.13

Variables summarised as mean±SD were compared using one-way analysis of variance. Variables that were significantly different between controls and participants with SLE are denoted as *p≤0.01, **p≤0.001 and ***p≤0.0001, †p≤0.05.

CNBD, corneal nerve branch density; CNFD, corneal nerve fibre density; CNFL, corneal nerve fibre length; CNFT, corneal nerve fibre tortuosity; DN4, Douleur Neuropathique 4; dsDNA, double-stranded DNA; ESC, electrochemical skin conductance; IWL, inferior whorl length; NA, not applicable; VPT, vibration perception threshold.

### Neuropathic pain patterns

According to the McGill Pain Questionnaire, 20.7% of participants reported transient neuropathic pain, 62.1% experienced recurrent pain and 17.2% reported sustained pain (table 3). There were no significant differences in anti-dsDNA, C3 or C4 levels between the pain groups. Steroid use was significantly higher in patients with sustained pain than in those with transient pain (70% vs 25%, p<0.05), whereas immunosuppressant use was comparable between the groups (p=0.28–1.00).

**Table 3:** Comparison of clinical, neuropathy and quality-of-life measures between different neuropathic pain patterns in SLE

	Controls (n=17)	Pain frequency in patients with SLE			P value^1^	P value^2^	P value^3^
		Transient	Recurrent	Sustained			
N (%)	17	12 (20.7)	36 (62.1)	10 (17.2)	N/A	N/A	N/A
Age, years	34.0±7.5	44.7±8.9*	37.3±7.0	37.8±14.6	0.01	0.07	0.88
Anti-dsDNA level (IU/mL)	N/A	28.0±45.1	29.3±58.3	8.0±12.9	0.95	0.37	0.25
C3 level (g/L)	N/A	1.1±0.3	4.5±20.8	11.6±33.2	0.64	0.27	0.36
C4 level (g/L)	N/A	0.2±0.1	1.4±6.8	1.6±4.3	0.56	0.58	0.91
Immunosuppressants, n (%)	N/A	7/12 (58.3)	21/36 (58.3)	7/9 (77.8)	1.00	0.35	0.28
Steroids, n (%)	N/A	3/12 (25.0)	16/36 (44.4)	7/10 (70.0)	0.23	<0.05	0.15
CNFD, fibres/mm^2^	34.0±6.9	33.1±8.7	32.9±6.5	25.9±4.4***	0.91	<0.05	<0.01
CNBD, branches/mm^2^	72.1±29.4	46.9±27.9*	43.1±19.2***	32.2±16.2***	0.63	0.14	0.19
CNFL, mm/mm^2^	24.2±4.4	19.4±5.4*	19.2±3.9***	15.6±3.1***	0.91	<0.05	<0.05
IWL, mm/mm^2^	35.6±5.9	43.7±6.8*	38.8±7.1	32.4±8.6	<0.05	<0.0001	0.01
CNFT	14.3±3.1	16.2±4.0	14.5±3.6	15.9±4.7	0.17	0.85	0.29
CNBD/CNFD ratio	2.07±0.57	1.37±0.73*	1.33±0.57***	1.24±0.63**	0.82	0.61	0.69
IWL/CNFL ratio	1.49±0.28	2.49±0.98***	2.09±0.55**	2.07±0.40*	<0.05	0.10	0.94
DN4 Questionnaire, Score	0.0±0.0	2.3±2.5*	3.8±2.5***	4.2±2.2***	<0.05	<0.05	0.61
VPT, V	2.8±0.7	5.8±5.9*	3.2±1.7	4.1±3.7	0.01	0.21	0.41
Sudomotor function of the hands, ESC	67.4±15.5	61.4±19.4	60.8±21.7	70.8±10.0	0.92	0.25	0.14
Sudomotor function of the feet, ESC	76.5±12.7	67.2±16.1	71.9±16.9	75.6±11.7	0.36	0.20	0.50
Physical functioning, %	94.4±11.2	84.2±22.7	75.0±20.8**	66.0±14.3***	0.15	<0.05	0.18
Limitations due to physical health problems, %	92.6±14.7	83.3±19.5	53.5±41.9***	50.0±33.3*	<0.01	<0.05	0.77
Limitations due to emotional problems, %	98.0±8.1	86.1±30.0	56.5±40.5***	40.0±46.6***	0.01	<0.01	0.19
Energy/fatigue, %	60.4±11.9	60.7±13.2	47.8±15.1*	44.3±18.1*	0.01	0.01	0.50
Emotional well-being, %	73.4±14.1	79.7±16.1	62.7±23.1	53.6±21.3*	0.01	<0.01	0.21
Social functioning, %	96.3±9.6	86.5±21.6	64.9±26.7***	56.3±28.4***	<0.01	<0.01	0.30
Bodily pain, %	90.0±15.4	82.3±17.8	61.5±22.5***	52.8±17.4***	<0.01	0.001	0.22
General health, %	74.1±16.9	66.7±15.6	54.3±22.2**	53.0±22.1*	0.07	0.12	0.86

Variables summarised as mean±SD were compared using one-way analysis of variance. P value1: value for the comparison between SLE with transient versus recurrent pain; P value2: value for SLE with transient versus sustained pain; and P value3: value for SLE with recurrent versus sustained pain. Variables that were significantly different between healthy controls and patients with SLE are denoted as *p≤0.01, **p≤0.001, ***p≤0.0001, †p≤0.05.

CNBD, corneal nerve branch density; CNFD, corneal nerve fibre density; CNFL, corneal nerve fibre length; CNFT, corneal nerve fibre tortuosity; DN4, Douleur Neuropathique 4; dsDNA, double-stranded DNA; ESC, electrochemical skin conductance; IWL, inferior whorl length; N/A, not applicable; VPT, vibration perception threshold.

### Corneal nerve fibre morphology

The SLE group had significantly lower CNBD (41.5±21.3 vs 72.1±29.4 branches/mm²), CNFL (18.5±4.3 vs 24.2±4.4 mm/mm²) and CNBD/CNFD ratio (1.30±0.62 vs 2.07±0.57) (all p=0.0001) and a higher IWL/CNFL ratio (p<0.0001) than healthy controls. CNFD (31.7±7.1 and 34.0±6.9, respectively, p=0.25), IWL (p=0.16) and corneal nerve fibre tortuosity (p=0.55) were comparable between the groups. As a sensitivity analysis, we repeated these comparisons after excluding the youngest three control participants (two male and one female) to increase the mean age and achieve a comparable sex distribution to the SLE group (online supplemental table 1). The differences in CNBD, CNFL, CNBD/CNFD and IWL/CNFL between controls and SLE participants remained statistically significant.

**Figure 1:**
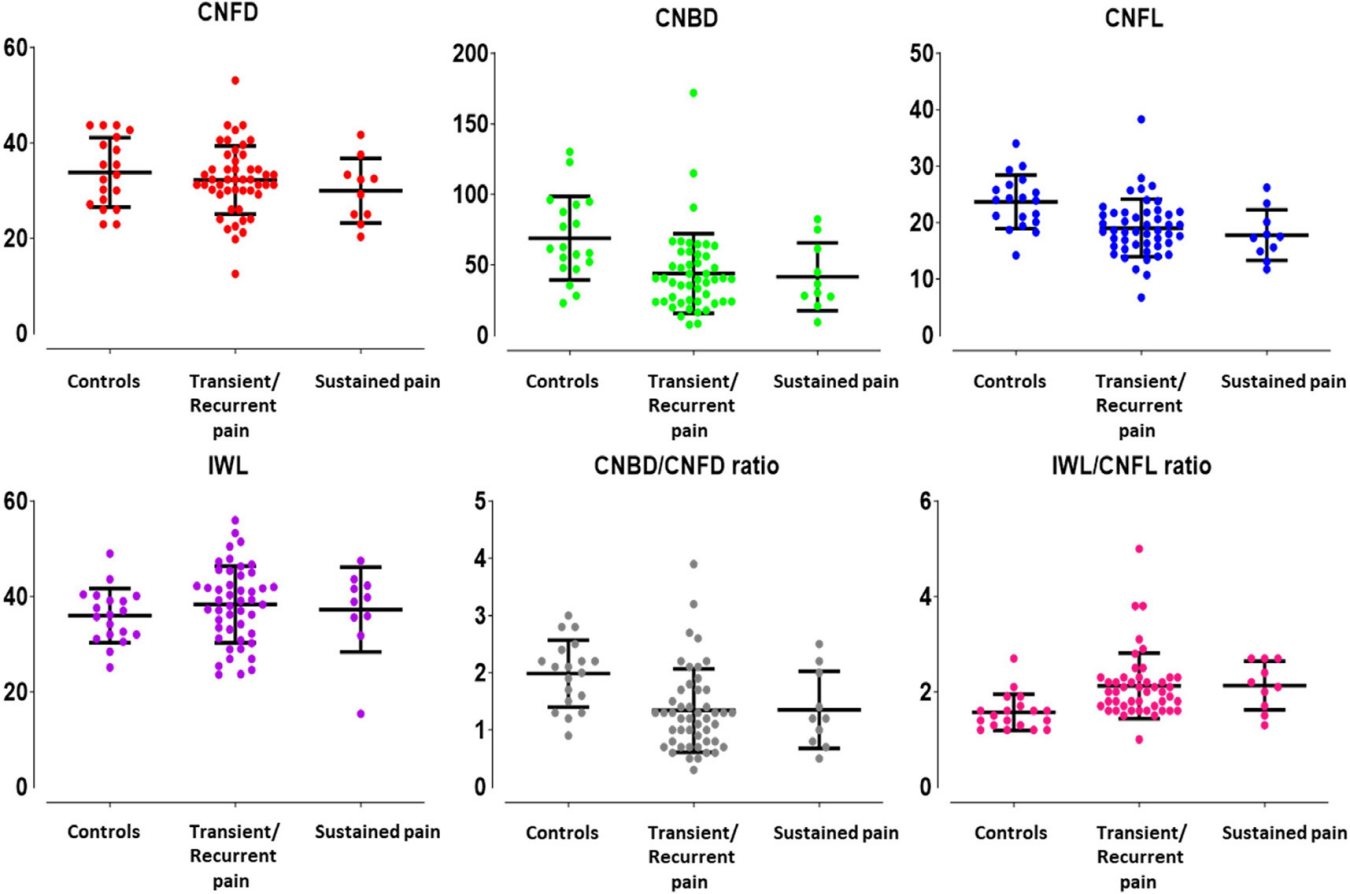
Comparison of corneal nerve fibre morphology between different pain frequencies in patients with SLE. Dot plots of corneal nerve fibre density (CNFD) (red), corneal nerve branch density (CNBD) (green), corneal nerve fibre length (CNFL) (blue), inferior whorl length (IWL) (purple), CNBD/CNFD ratio (grey) and IWL/CNFL ratio (violet) in healthy controls, patients with SLE associated with transient or recurrent pain, and patients with SLE associated with sustained pain are demonstrated. The lines that extend from the middle of the vertical line represent the mean and the lines that extend to the top and bottom represent the SD, with significant differences observed among the three groups.*p≤0.01; **p≤0.001; ***p<0.0001; ‡p≤0.05.

Patients with active SLE had a significantly lower CNBD/CNFD ratio than those with inactive SLE (1.17±0.56 vs 1.53±0.65, p<0.05) (table 2). Patients with SLE with sustained pain had significantly lower CNFD, CNFL and IWL values than those with transient pain (p<0.05–0.0001) and recurrent pain (p<0.05–0.01) (table 3; figures1  2). Corneal nerve morphology was comparable between patients with and without SLE nephritis (n=17/59, 28.8% and n=42/59, 71.2%, respectively; p=0.52–0.84).

**Figure 2:**
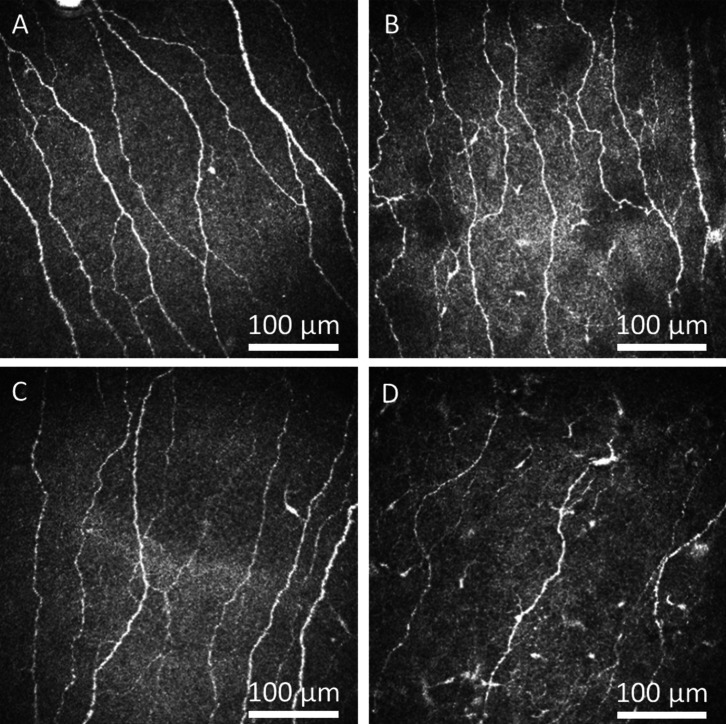
Representative corneal confocal microscopy images of the sub-basal nerves in a healthy control and in patients with SLE associated with different pain patterns. Corneal nerve fibres in (**A**) healthy controls, (**B**) a patient with SLE associated with transient pain, (**C**) a patient with SLE associated with recurrent pain and (**D**) a patient with SLE associated with sustained pain showing progressive reduction in corneal nerve fibres.

### Neuropathic symptoms and deficits

Compared with healthy controls, participants with SLE had a mean DN4 Score of 3.5±2.5 and a significantly higher VPT (4.1±3.3 vs 2.8±0.7 V, p<0.01) but within the normal range of 1–15 V. Sudomotor functions of the hands and feet were comparable between the groups (p=0.28–0.42). Neuropathic signs and symptoms, VPT and sudomotor function were comparable between patients with and without active SLE (p=0.12–0.27) (table 2). In the same sensitivity analysis, DN4 Score differences remained statistically significant, whereas VPT lost significance (p=0.06) (online supplemental table 1).

Compared with patients with transient pain, those with sustained pain had significantly higher rates of neuropathic symptoms (p<0.05) but comparable VPT (p=0.27) (table 3). Patients with recurrent pain had higher rates of neuropathic symptoms (p<0.05) and VPT (p=0.01) than those with transient pain. Sudomotor function was comparable between patients with transient, recurrent and sustained pain (p=0.14–0.92).

### Quality of life: SF-36 Questionnaire

Compared with healthy controls, patients with SLE reported greater difficulty with physical activities, more limitations in their daily roles due to physical health concerns, increased restrictions caused by emotional issues, higher levels of fatigue, diminished social interaction, more intense pain and a lower overall perception of general health (p<0.01–0.0001). The average percentage of all six SF-36 domains, including restrictions due to physical health concerns (p<0.05–0.01), emotional issues (p≤0.01), fatigue (all p=0.01), difficulty with emotional well-being (p≤0.01), diminished social interaction (all p<0.01) and pain (all p<0.01), was significantly lower in patients with recurrent and sustained pain than in those with transient pain (table 3).

Bodily pain was significantly associated with reduced CNFL (β=1.24%/mm/mm², 95% CI 0.18% to 2.3%, p<0.05). In the bodily pain model, haemoglobin was also a significant covariate (β=4.56%/g/dL, 95% CI 1.19% to 7.93%, p≤0.01). The association of physical functioning, role limitations due to physical health, emotional issues, fatigue, diminished social interaction, and general health with reduced CNBD and CNFL was not significant after adjusting for age and haemoglobin in the physical functioning model, and for haemoglobin in the other models. None of the eight domains of SF-36 were associated with VPT, sudomotor function, CNFD, CNFL or IWL.

## Discussion

Our findings revealed corneal small nerve fibre degeneration with normal sudomotor function in patients with SLE, indicating that CCM is a sensitive technique for early detection of subclinical SFN. Furthermore, evidence of reduced small nerve fibre regeneration (CNBD/CNFD) in active SLE and greater corneal nerve loss in patients with sustained neuropathic pain and bodily pain affecting the quality of life was observed.

Corneal nerve degeneration has been demonstrated previously in other neurological autoimmune conditions, such as multiple sclerosis^26 27^ and Sjögren’s syndrome.^29^ Reductions in CNBD and CNFL, but not CNFD, underscore the importance of a comprehensive assessment of all corneal nerve parameters when using CCM. Furthermore, IWL was considerably higher than CNFL, suggesting proximal corneal nerve involvement in patients with SLE.

Active SLE is associated with peripheral nerve damage.^6 8^ Indeed, Bitirgen *et al* reported that active SLE is associated with greater corneal nerve degeneration than inactive SLE.^33^ In the current study, CNFD, CNBD and CNFL were comparable; however, the CNBD/CNFD ratio was higher in patients with active SLE than in those with inactive SLE, indicating impaired regeneration. Furthermore, we observed greater corneal nerve degeneration in patients with SLE associated with sustained neuropathic pain than in those with recurrent or transient neuropathic pain, highlighting the importance of early detection and intervention in patients with SLE associated with persistent pain. Omdal *et al* and Gøransson *et al* also demonstrated small nerve fibre involvement in SLE.^8 9^ Moreover, Oomatia *et al* reported an association between peripheral neuropathies and SLE, particularly in patients with chronic pain syndromes.^6^ Tekatas *et al* highlighted the utility of cutaneous silent periods and skin biopsies in the assessment of early SFN in SLE, although these techniques are complex to perform and analyse.^10^ It is important to note that high disease activity in this cross-sectional study may also serve as a marker of more severe SLE, which in turn could lead to increased cumulative treatment exposure. These treatments themselves can contribute to nerve fibre damage,^43^,^45^ making it challenging to distinguish the effects of active inflammation from treatment-related neurotoxicity.

We found that reduced CNFL was associated with bodily pain and reduced quality of life in patients with SLE. Indeed, Monahan *et al* reported that neuropsychiatric symptoms and neuropathic pain in SLE considerably affect patients’ quality of life.^5^ Brey *et al* highlighted the high prevalence of neuropsychiatric syndromes in SLE and their profound impact on daily functioning,^3^ whereas Unterman *et al* demonstrated the detrimental effect of neuropsychiatric syndromes in SLE on mental and physical health.^4^ The role of sustained pain and small nerve fibre damage in patients with impaired quality of life suggests that interventions to prevent small fibre damage could mitigate neuropathic pain and improve quality of life.

Of note, this is a cross-sectional study and causality cannot be established. The association between SLE and small fibre neuropathy might indicate a common pathogenic process, also possibly including immune dysregulation, rather than causality.^46 47^ Socioeconomic and environmental exposures may also independently contribute to both conditions; for example, lower socioeconomic condition is associated with more severe lupus outcomes, most likely through barriers to healthcare access and environmental triggers that increase the risk of developing neuropathy.^48 49^ Finally—perhaps most relevant—treatment-related neurotoxicity remains a plausible contributor: antimalarials are linked to neuromyotoxicity,^43^ calcineurin inhibitors can cause polyneuropathy^44^ and rare cases have been reported with mycophenolate—making cumulative treatment exposure an important consideration in interpreting small fibre findings.^45^ Prospective studies capturing longitudinal disease activity and detailed cumulative treatment data are needed to disentangle these effects.

Our study provides valuable insights into the use of CCM in detecting small nerve fibre abnormalities in participants with SLE, but it has some limitations. The sample size was small; thus, larger studies are required to validate our findings. In addition, the cross-sectional design limited our ability to draw causal inferences. Hence, longitudinal studies are warranted to assess the changes in corneal nerve parameters over time and their relationship with disease progression and treatment response. Furthermore, although we excluded patients with overt dry eye using the Schirmer test, we did not use the Ocular Surface Disease Index Questionnaire or the tear break-up time test. While our analysis focused on quantitative measures of corneal nerve morphology, we also measured microneuromas. These will be analysed separately in future work, as they may have distinct associations with other aspects of SLE. Hyper-reflective epithelial patches, another qualitative feature described in neuropathic corneal pain,^50^ were not assessed in the present study but could provide complementary information in future analyses.

In conclusion, our study shows that CCM can detect small nerve fibre changes in patients with SLE, which are associated with disease activity, neuropathic symptoms and reduced quality of life. Although these findings suggest the potential utility of CCM as a rapid, non-invasive neuroimaging biomarker in assessing small fibre involvement in SLE, further longitudinal studies with confirmed SFN are warranted to validate its clinical relevance as well as assess the long-term impact and the potential benefits of early therapeutic interventions aimed at protecting small nerve fibres in patients with SLE.

## Supplementary material

10.1136/lupus-2025-001645 online supplemental table 1

## Data Availability

Data are available upon reasonable request.
