# Supplementary material for: Corneal confocal microscopy: a novel biomarker of small fibre neuropathy in SLE
Source: Lupus Sci Med. 2025 Oct 23;12(2):e001645. doi: 10.1136/lupus-2025-001645 (PMC12551467; doi:10.1136/lupus-2025-001645)
Supplement: online supplemental table 1 [file lupus-12-2-s001.docx]

Supplementary Table 1. Comparison of neuropathy between patients with systemic lupus erythematosus and healthy controls after removal of the three youngest controls.

|  | Controls (n=14) | Patients with SLE (n=59) | P-value |
| --- | --- | --- | --- |
| Age, years | 34.7±7.8 | 38.6±9.6 | 0.16 |
| Female sex, n (%) | 12 (85.7) | 56 (94.9) | 0.22 |
| CNFD, fibres/mm^2^ | 35.1±6.8 | 31.7±7.1 | 0.11 |
| **CNBD, branches/mm^2^** | 72.1±31.6 | 41.5±21.3 | **<0.0001** |
| **CNFL, mm/mm^2^** | 24.7±4.6 | 18.5±4.3 | **<0.0001** |
| IWL, mm/mm^2^ | 36.0±6.3 | 38.5±8.0 | 0.28 |
| CNFT | 14.5±3.2 | 15.0±4.0 | 0.69 |
| **CNBD/CNFD ratio** | 1.98±0.57 | 1.30±0.62 | **<0.0001** |
| **IWL/CNFL ratio** | 1.49±0.28 | 2.16±0.64 | **<0.0001** |
| **DN4 questionnaire, score** | 0±0 | 3.5±2.5 | **<0.0001** |
| **VPT, Volts** | 2.9±0.7 | 4.1±3.3 | 0.06 |
| Sudomotor function of the hands, ESC | 65.0±16.2 | 63.0±19.9 | 0.74 |
| Sudomotor function of the feet, ESC | 74.9±13.6 | 71.8±16.0 | 0.52 |

Variables summarised as mean ± standard deviation were compared using the independent t-test. Abbreviations: corneal nerve fibre density (CNFD), corneal nerve branch density (CNBD), corneal nerve fibre length (CNFL), corneal nerve fibre tortuosity (CNFT), inferior whorl length (IWL), vibration perception threshold (VPT), and systemic lupus erythematosus (SLE).
